# Did the prolonged residual efficacy of clothianidin products lead to a greater reduction in vector populations and subsequent malaria transmission compared to the shorter residual efficacy of pirimiphos-methyl?

**DOI:** 10.1186/s12936-024-04949-4

**Published:** 2024-04-25

**Authors:** Esdras Mahoutin Odjo, Christian S. T. Akpodji, Armel Djènontin, Albert Sourou Salako, Gil Germain Padonou, Constantin Jésukèdè Adoha, Boulais Yovogan, Bruno Adjottin, Filémon T. Tokponnon, Razaki Osse, Clement Agbangla, Martin C. Akogbeto

**Affiliations:** 1 Centre de Recherche Entomologique de Cotonou, Cotonou, Bénin; 2 Faculté des Sciences et Techniques, Université d’Abomey- Calavi, Calavi, Bénin; 3 Université Nationale d’Agriculture de Porto-Novo, Porto-Novo, Bénin; 4 Direction Générale de la Recherche Scientifique, Ministère de l’Enseignement Supérieur et de la Recherche Scientifique, Cotonou, Bénin

**Keywords:** Malaria, IRS, Vector control, Pirimiphos-methyl, Clothianidin alone, Clothianidin and deltamethrin, Entomological indicators

## Abstract

**Background:**

The residual activity of a clothianidin + deltamethrin mixture and clothianidin alone in IRS covered more than the period of malaria transmission in northern Benin. The aim of this study was to show whether the prolonged residual efficacy of clothianidin-based products resulted in a greater reduction in vector populations and subsequent malaria transmission compared with the shorter residual efficacy of pirimiphos-methyl.

**Methods:**

Human bait mosquito collections by local volunteers and pyrethrum spray collections were used in 6 communes under IRS monitoring and evaluation from 2019 to 2021. ELISA/CSP and species PCR tests were performed on *Anopheles gambiae *sensu lato (*s.l*.) to determine the infectivity rate and subspecies by commune and year. The decrease in biting rate, entomological inoculation rate, incidence, inhibition of blood feeding, resting density of *An. gambiae s.l.* were studied and compared between insecticides per commune.

**Results:**

The *An*. *gambiae* complex was the major vector throughout the study area, acounting for 98.71% (19,660/19,917) of all *Anopheles* mosquitoes collected. *Anopheles gambiae s.l.* collected was lower inside treated houses (45.19%: 4,630/10,245) than outside (54.73%: 5,607/10,245) after IRS (p < 0.001). A significant decrease (p < 0.001) in the biting rate was observed after IRS in all departments except Donga in 2021 after IRS with clothianidin 50 WG. The impact of insecticides on EIR reduction was most noticeable with pirimiphos-methyl 300 CS, followed by the clothianidin + deltamethrin mixture and finally clothianidin 50 WG. A reduction in new cases of malaria was observed in 2020, the year of mass distribution of LLINs and IRS, as well as individual and collective protection measures linked to COVID-19. *Anopheles gambiae s.l.* blood-feeding rates and parous were high and similar for all insecticides in treated houses.

**Conclusion:**

To achieve the goal of zero malaria, the optimal choice of vector control tools plays an important role. Compared with pirimiphos-methyl, clothianidin-based insecticides induced a lower reductions in entomological indicators of malaria transmission.

## Background

The reduction in malaria incidence by 18% worldwide between 2010 and 2016, and by 20% in the WHO African Region [1], was the result of the intensive use of long-lasting insecticidal nets (LLINs) and indoor residual spraying (IRS) [2]. IRS is an important intervention that can rapidly reduce the density and longevity of disease-carrying mosquitoes when carried out properly [3]. It has had a positive impact on entomological transmission indicators (ETI) of malaria in Benin since 2008 in sprayed communes [4]. Similarly, in several other African countries, IRS has reduced malaria transmission and burden in children [5–9]. However, despite the progress made in implementing vector control and treatment access strategies, challenges remain, and the decline in malaria morbidity and mortality has slowed in recent years [10]. Several hypotheses have attempted to explain the slowdown in progress after 2016. The lack of predictable, sustainable and solid funding at national and international level to ensure continuous surveillance, and the resistance of mosquitoes to insecticides, could be major causes of the stagnation of progress [11, 12].

To advance progress and achieve the goals of reducing global malaria mortality rates to minus 75% by 2025, reducing global malaria incidence to minus 75% by 2025, eliminating malaria from 20 countries with transmission in 2015 by 2025, and preventing the re-emergence of malaria in malaria-free countries, it is important to overcome the difficulties associated with poorly functioning health systems, in particular the use of ineffective vector control tools [12]. Indeed, the selection of an insecticide product for IRS should always be a decision made on the basis of recent local data on the susceptibility of target vectors to insecticides [13]. However, to optimize the impact of IRS and to manage potential resistance of malaria vectors to pirimiphos-methyl (after three years of IRS use), Benin's National Malaria Control Programme (NMCP) opted for the use of clothianidin 500 g/kg + deltamethrin 62.5 g/kg and clothianidin 50 WG alone in 2020 and 2021, respectively. Clothianidin 50 WG and the mixture clothianidin 500 g/kg and deltamethrin 62.5 g/kg are two new IRS formulations prequalified by the WHO in 2017 and 2018 [14]. Clothianidin is a neonicotinoid that targets nicotinic acetylcholine receptors, a representing a novel target for public health vector control interventions. In Benin, clothianidin-based insecticides have a long residual efficacy of 8 to 10 months in large-scale in community trials on mud and cement walls, in contrast to pirimiphos-methyl 300 CS, which has a residual efficacy of 4 to 5 months [15].

The aim of the present study was to show whether the prolonged residual efficacy of clothianidin-based products resulted in a greater reduction in vector populations and subsequent malaria transmission compared with the shorter residual efficacy of pirimiphos-methyl. The findings at the end of the evaluation will enable decision-makers to make a judicious choice of insecticide for indoor residual spraying, taking into account not only the cost-effectiveness of the insecticide with the best residual activity, but also the residual activity of the insecticide on walls and its impact in reducing malaria ETIs.

## Methods

### Study area

**Fig. 1:**
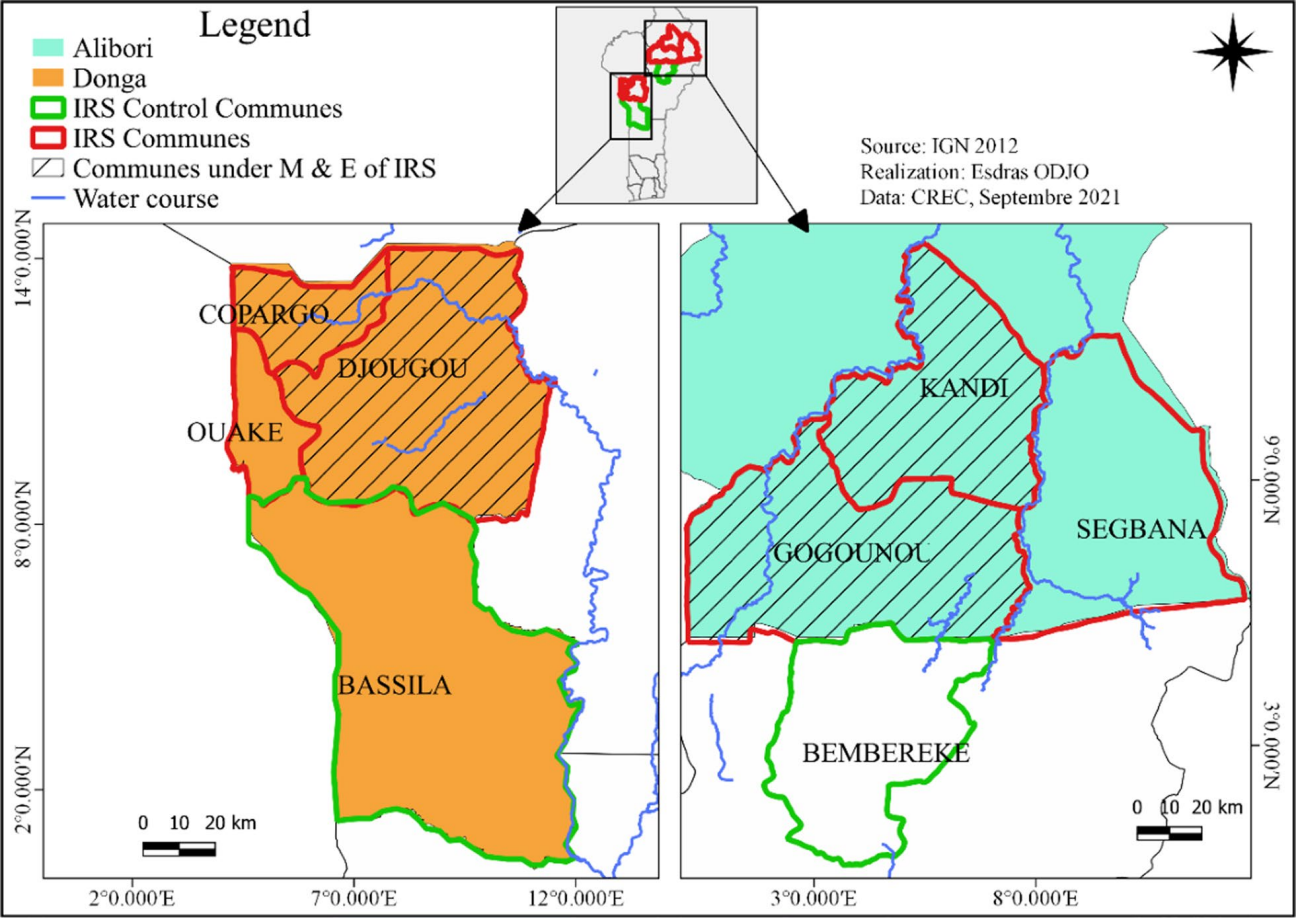
Map of the study area

Djougou, Ouaké, Copargo (DOC) health zone (ZS) in Donga department and Kandi, Gogounou, Segbana (DGS) in Alibori department were covered by IRS during the study period (Fig. 1). A total of 6 communes, including 4 (Djougou, Copargo, Kandi and Gogounou) under IRS coverage and 2 control communes not treated with insecticide (Bassila in Donga department and Bembèrèkè in Alibori department) were selected for entomological monitoring and evaluation (M&E). The control communes were chosen on the basis of their proximity to, and similar climatic conditions to, the communes sprayed under the M&E programme.

The NMCP of Benin has adopted a policy of mass distribution ITNs since 2011, on a triennial basis as recommended by the WHO. The 4th mass distribution campaign took place in 2020 and has been digitized. 699,957 (95.94%) and 407,922 (89.19%) ITNs were distributed in Alibori and Donga, respectively [16].

Each commune is characterized by 2 seasons. A rainy season corresponding to the period after IRS when malaria transmission increases, and a dry season. During the dry season, mosquito density drops considerably, with little or no contact between man and vector, and consequently low malaria transmission. Indoor residual spraying campaigns are carried out at the end of the dry season [15] with the aim of reducing peak transmission following the increase in rains. Annual rainfall from 2019 to 2021 ranged from 970 to 1007 mm in Alibori and from 1147 to 1150 mm in Donga [17].

### Mosquito sampling methods

Mosquitoes were sampled in the 6 communes selected for IRS monitoring and evaluation in 2019, 2020 and 2021, using human landing catches (HLCs) by local volunteers and collection of mosquitoes by pyrethrum spray catch (PSC) with non-residual insecticide, every month for 10 months.

For each sampling method, mosquitoes were collected in two villages per commune, one in the center and one on the outskirts of the commune. Within each village, mosquitoes were collected by HLC in 4 houses from 7 pm to 7am. Two teams of eight mosquito collectors were set up in each village. The first team worked inside and outside the selected dwellings from 19:00 to 01:00, and the second team from 01:00 to 07:00. In order to avoid biases linked to the trapping capability of the catchers or their individual attractiveness, they were rotated between the collection locations. Collections were carried out using haemolysis tubes and cotton. Mosquitoes collected were sorted by site, capture location and time of day.

In addition, 10 dwellings were selected per village on the eve of each collection by PSC to calculate the average indoor resting density of mosquitoes. Early in the morning, after the household members had exited, the dwellings were sprayed with pyrethrum and kept closed for 10 to 15 min. A white cloth was laid out on the floor to collect any mosquitoes that fell. At the end of the allotted time, all the mosquitoes that had fallen to the ground were collected and placed in Petri dishes. The number of mosquitoes per room and their blood-feeding stage (blood fed, half-gravid, gravid and unfed) were assessed.

### Morphological and molecular identification of vector species

Mosquitoes collected by HLC and PSC were morphologically identified using Gillies & De Meillon’s taxonomic key [18] and recorded by village (central, peripheral), site (house 1 to house 10), location of capture (indoor, outdoor) and time. Malaria vector specimens were stored individually in cryoboxes.

The abdomen, legs and wings of *Anopheles gambiae *sensu lato (*s.l.)* captured by HLC were analysed by PCR according to the protocol of Santolamazza et al*.* [19] for molecular characterization of the species of the complex.

### Parous and *Plasmodium falciparum* infection

A sample of *An. gambiae s.l.* captured by local volunteers was dissected and tracheoles examined [20] to determine the physiological age of malaria vectors in the various departments.

The heads-thoraxes of all females of the vector species were analysed by ELISA for circumsporozoite antigen (CSP) according to the protocol described by Wirtz et al. [21].

### Epidemiological data collection

During the three years of the study, new cases of uncomplicated and severe malaria tested positive by thick blood smears (TBS) and rapid diagnostic test (RDT) were counted in the health zones (ZS) by communes under IRS coverage. Data from control communes not targeted for IRS were also collected. These data were used to assess the incidence of malaria in the study areas.

### Entomological transmission parameters

Entomological parameters were compared for data collected from spray campaigns with pirimiphos-methyl 300 CS (2019) and clothianidin insecticides (2020 and 2021).

Biting rate per man per night (HBR), sporozoite rate (SR), entomological inoculation rate (EIR) per man per night, resting density of vectors, parous rate, blood-feeding rate were calculated for *An. gambiae s.l*. following World Health Organization (WHO) guidance [22].

### Data analysis

Field and laboratory data were entered into Excel 2013 and analysed using R statistical software, version 4.1.3. Comparisons were made within each department, overall between treated communes and between treated and control communes. The Chi-square test for comparison of proportions was used to define relationships between areas under IRS coverage by year and the following indicators: proportion of *An. gambiae s.l.* indoors and outdoors, blood feeding rate, *An. gambiae s.l.* parous rate, sporozoite rate. The Poisson test was used to estimate the rate ratio (RR) and the confidence intervals of indoor vector density and the EIR of *An. gambiae s.l..*

## Results

### Composition of mosquitoes collected

Overall, 50,645 mosquitoes of 4 genera and 19 species were collected in 4 IRS communes and 2 control communes under IRS monitoring from 2019 to 2021. *Culex quinquefasciatus* was the most commonly captured species (58.5%), followed by *An. gambiae s.l.* (38.8%) and *Mansonia africana* (0.9%). *Anopheles* mosquitoes accounted for 39.3% of mosquitoes obtained by HLC. The *An. gambiae* complex and the *Anopheles funestus* group accounted for 98.7% and 1%, respectively. The other *Anopheles*, namely: *Anopheles broheri*; *Anopheles coustani*; *Anopheles nili*; *Anopheles paludis*; *Anopheles pharoensis* and *Anopheles ziemanni* accounted for 0.36%. (Table 1).

**Table 1:** Species composition of mosquitoes collected in 2019, 2020 and 2021

Species	2019	2020	2021	Total	Proportion (%)
*An. gambiae*	4572	5372	9716	19660	38.8
*An. funestus*	29	84	77	190	0.4
*An. broheri*	0	0	9	9	0.0
*An. coustani*	0	3	1	4	0.0
*An. nili*	0	4	1	5	0.0
*An. paludis*	2	0	0	2	0.0
*An. pharoensis*	10	15	7	32	0.1
*An. ziemanni*	3	2	10	15	0.0
*Cx. quinquefasciatus*	11272	12246	6106	29624	58.5
*Cx. tigripes*	9	4	4	17	0.0
*Cx. descens*	25	6	10	41	0.1
*Cx. nebulosus*	45	81	158	284	0.6
*Aedes aegypti*	89	105	60	254	0.5
*Aedes albopictus*	0	0	1	1	0.0
*Aedes longipalpis*	0	0	7	7	0.0
*Aedes luteocephalus*	5	3	7	15	0.0
*Aedes vittatus*	23	1	20	44	0.1
*Mansonia africana*	114	218	101	433	0.9
*Mansonia uniformis*	1	2	5	8	0.0
Total	16199	18146	16300	50645	100

%: percentage; *Cx*.: *Culex*; *An*.: *Anopheles*

**Fig. 2:**
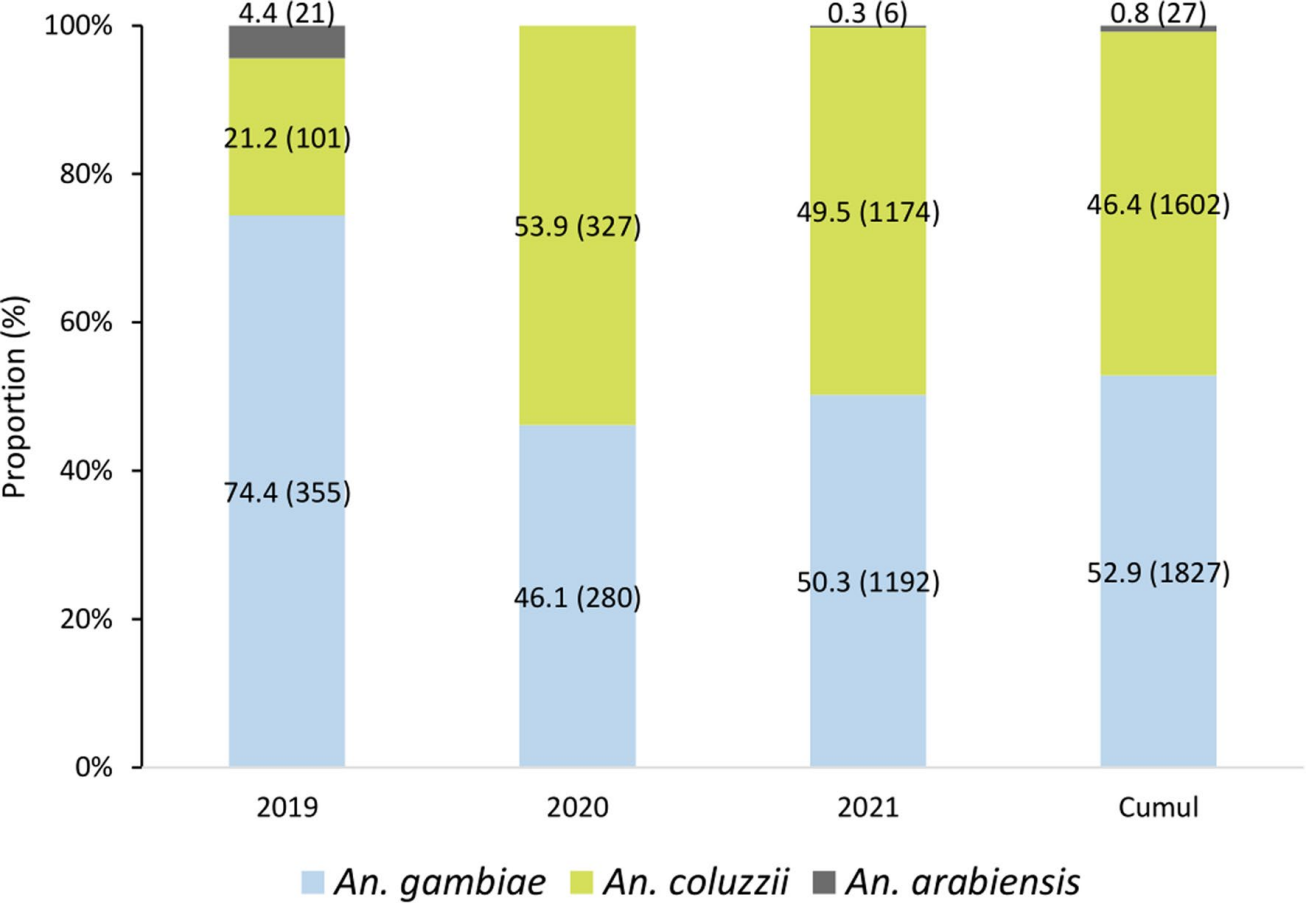
Composition of *An. gambiae s.l.* subspecies in IRS communes and control communes from 2019 to 2021

Over the three years, a total of 3,456 *An*. *gambiae s.l.* were analysed by PCR for molecular species identification: 1,602 (46.4%) *Anopheles coluzzii*, 1,827 (52.86%) were *An. gambiae *sensu stricto* (s.s.),* and 27 *Anopheles arabiensis* (0.78%) (Fig. 2). *Anopheles gambiae* and *An*. *coluzzii* were detected in similar proportions in 2020 and 2021 while in 2019, *An*. *gambiae* predominated (74.4%).

### Biting rate of *An. gambiae* inside and outside houses

#### Alibori (IRS communes: Kandi + Gogounou; commune control: Bembèrèkè)

The rate ratio of human bites per night before and after implementation of IRS inside and outside dwellings in the control commune (RR1: 12.55–30.41) is higher than in the communes subject to IRS (RR2: 2.75–7.09) (Table 2). The mean human biting rates (HBR) of *An. gambiae* estimated was lower inside treated houses than outside after IRS (P2 < 0.05), except in 2020 with the mixture clothianidin 500 g/kg + deltamethrin 62.5 g/kg (Table 2). In homes not treated with insecticides (Controls), biting rates were higher indoors before and after IRS. However, in 2021 after IRS with clothianidin 50 WG, bite rates were similar inside and outside the untreated houses (1001/32 and 1063/32, p-value = 0.18).

**Table 2:** Number and proportion of *An. gambiae s.l.* collected indoors and outdoors in treated communes (Kandi, Gogounou, Djougou, Copargo) and control communes (Bembèrèkè, Bassila) in 2019, 2020 and 2021

Location	Insecticide (Year)	Period	Department of Alibori					Department of Donga				
			Control Commune (Bembèrèkè)		Communes under IRS (Kandi + Gogounou)		P3 (RR3)	Control Commune (Bassila)		Communes under IRS (Djougou + Copargo)		P3 (RR3)
			HBR/night (Eff/NbC)	P1 (RR1)	HBR/night (Eff/NbC)	P2 (RR2)		HBR/night (Eff/NbC)	P1 (RR1)	HBR/night (Eff/NbC)	P2 (RR2)	
Indoors	PM 300 CS (2019)	Before IRS	1.6 (51/32)	< 0.0001 (19.65)	1.9 (120/64)	< 0.0001 (2.75)	0.372 (1.18)	1.1 (35/32)	< 0.0001 (9.77)	0.7 (45/64)	< 0.0001 (7.76)	0.0572 (0.64)
		After IRS	31.3 (1002/32)		5.2 (330/64)		< 0.0001 (0.16)	10.7 (342/32)		5.5 (349/64)		< 0.0001 (0.51)
	Clo + Del (2020)	Before IRS	1.0 (16/16)	< 0.0001 (12.55)	2.0 (97/48)	< 0.0001 (7.09)	0.0064 (2.02)	0.8 (13/16)	< 0.0001 (30.34)	0.9 (44/48)	< 0.0001 (22.66)	0.7622 (1.13)
		After IRS	26.1 (521/20)		14.3 (573/40)		< 0.0001 (0.55)	24.7 (493/20)		20.8 (831/40)		0.0029 (0.84)
	Clo 50 WG (2021)	Before IRS	1.6 (39/24)	< 0.0001 (19.25)	5.5 (262/48)	< 0.0001 (3.29)	< 0.0001 (3.36)	1.5 (37/24)	< 0.0001 (11.74)	1.4 (68/48)	< 0.0001 (15.48)	0.6797 (0.92)
		After IRS	31.3 (1001/32)		18.0 (1151/64)		< 0.0001 (0.57)	18.1 (579/32)		21.9 (1404/64)		< 0.0001 (1.21)
Outdoors	PM 300 CS (2019)	Before IRS	0.8 (27/32)	< 0.0001 (30.41)	2.1 (136/64)	< 0.0001 (3.96)	< 0.0001 (2.52)	0.6 (19/32)	< 0.0001 (13.11)	0.5 (34/64)	< 0.0001 (13.94)	0.7709 (0.89)
		After IRS	25.7 (821/32)		8.4 (538/64)		< 0.0001 (0.33)	7.8 (249/32)		7.4 (474/64)		0.5281 (0.95)
	Clo + Del (2020)	Before IRS	0.8 (13/16)	< 0.0001 (20.43)	2.1 (101/48)	< 0.0001 (5.79)	0.0005 (2.59)	1.5 (24/16)	< 0.0001 (24.93)	0.8 (37/48)	< 0.0001 (33.79)	0.0169 (0.51)
		After IRS	16.6 (332/20)		12.2 (487/40)		< 0.0001 (0.73)	37.4 (748/20)		26.1 (1042/40)		< 0.0001 (0.70)
	Clo 50 WG (2021)	Before IRS	1.3 (30/24)	< 0.0001 (26.58)	4.5 (216/48)	< 0.0001 (4.49)	< 0.0001 (3.60)	1.8 (42/24)	< 0.0001 (12.61)	1.1 (52/48)	< 0.0001 (25.57)	0.0217 (0.62)
		After IRS	33.2 (1063/32)		20.2 (1293/64)		< 0.0001 (0.61)	22.1 (706/32)		27.7 (1773/64)		< 0.0001 (1.26)

Eff: Number of *Anopheles gambiae s.l*. collected by HLC; NbC: Number of catchers; RR: rate ratio Poisson test R 4.1.3; PM: pirimiphos-methyl; CS: capsule suspensions; WG: Water dispersible granules; Clot + Del: mixture clothianidin 500 g/kg + deltamethrin 62.5 g/kg; Clo 50 WG: clothianidin 50 WG alone; IRS: indoor residual spraying

RR1: Rate ratio of HBR per night before and after the implementation of IRS in control commune within each department, with before IRS period as the reference

P1: p-value of RR1 methods comparison

RR2: Rate ratio of HBR per night before and after the implementation of IRS in communes under IRS within each department, with before IRS period as the reference

P2: p-value of RR2 methods comparison

RR3: Rate ratio of HBR per night before and after the implementation of IRS between control commune and communes under IRS within each department, with control commune as the reference

P3: p-value of RR3 methods comparison

Overall, a significant reduction (P3 < 0.001) in the biting rate was observed after IRS indoors and outdoors between the treated and control communes (Table 2). This reduction was greater in 2019 with pirimiphos-methyl 300 CS (RR3 = 0.16 indoors and 0.33 outdoors). For clothianidin-based insecticides, the reduction rate of clothianidin 50 WG was slightly higher than that of clothianidin 500 g/kg + deltamethrin 62.5 g/kg. Reduction rates were 86.1%; 72.8%; 82.8% indoors and 87.1%; 71.7%; 83.1% outdoors for pirimiphos-methyl 300 CS, mixture clothianidin 500 g/kg + deltamethrin 62.5 g/kg and clothianidin 50 WG alone, respectively.

#### Donga (IRS communes: Djougou + Copargo; commune control: Bassila)

The mean HBR of *An. gambiae* estimated was lower inside (5.5; 20.8; 21.9) treated houses than outside (7.4; 26.1; 27.7) after IRS with pirimiphos-methyl 300 CS, mixture clothianidin 500 g/kg + deltamethrin 62.5 g/kg and clothianidin 50 WG alone (Table 2). Also, a reduction in the biting rate was observed inside houses treated with pirimiphos-methyl 300 CS and mixture clothianidin 500 g/kg + deltamethrin 62.5 g/kg. The ratio [(C1 × T2) / (C2 × T1)] > 1 [23] for clothianidin 50 WG alone indoors and outdoors suggests that no reduction occurred in the population from the treatment. The same observation was made with pirimiphos-methyl 300 CS and the mixture clothianidin 500 g/kg + deltamethrin 62.5 g/kg outside treated houses (Table 2). For the ratio [(C1 × T2) / (C2 × T1)]:

C1 = Before IRS for control commune;

T1 = Before IRS for treated communes;

C2 = After IRS for control commune;

T2 = After IRS for treated communes.

### Sporozoite rate (SR)

Table 3 summarizes the sporozoite rate (SR) recorded before and after IRS in 2019, 2020 and 2021 in treated and control communes. A total of 19,308 head-thoraxes of *An. gambiae* collected by HLC and PSC were analysed by ELISA-CSP. 10,251 head-thoraxes of *An. gambiae* (4879 in the control commune and 5372 in the treated communes) were analysed in Alibori department and 9057 (3140 in the control commune and 5917 in the treated communes) in Donga department.

**Table 3:** Sporozoite rate (SR) of *An. gambiae s.l.* in treated and control communes in Alibori and Donga departments in 2019, 2020 and 2021

Location	Insecticide (Years)	Period	Department of Alibori			Department of Donga		
			Control Commune (Bembèrèkè)	Communes under IRS (Kandi + Gogounou)	P	Control Commune (Bassila)	Communes under IRS (Djougou + Copargo)	P
			SR % (A/B)	SR % (A/B)		SR % (A/B)	SR % (A/B)	
Indoors	PM 300 CS (2019)	Before IRS	5.9 (3/51)	0.0 (0/120)	0.041	14.3 (5/35)	2.2 (1/45)	0.109
		After IRS	1.7 (17/1002)	0.0 (0/330)	0.036	7.0 (24/342)	0.6 (2/349)	< 0.0001
	Clo + Del (2020)	Before IRS	0.0 (0/16)	0.0 (0/97)	NA	0.0 (0/13)	2.3 (1/44)	1
		After IRS	1.2 (6/521)	0.4 (2/573)	0.230	3.0 (15/493)	1.0 (8/831)	0.010
	Clo 50 WG (2021)	Before IRS	0.0 (0/39)	0.6 (2/318)	1	2.1 (1/47)	1.0 (1/100)	1
		After IRS	1.7 (17/989)	1.5 (18/1177)	0.860	5.0 (27/542)	4.5 (61/1365)	0.7186
Outdoors	PM 300 CS (2019)	Before IRS	7.4 (2/27)	0.7 (1/136)	0.116	5.3 (1/19)	5.9 (2/34)	1
		After IRS	0.9 (7/821)	0.9 (5/538)	1	6.4 (16/249)	0.4 (2/474)	< 0.0001
	Clo + Del (2020)	Before IRS	0.0 (0/13)	0.0 (0/101)	NA	0.0 (0/24)	2.7 (1/37)	1
		After IRS	1.5 (5/332)	0.6 (3/487)	0.363	0.8 (6/748)	0.6 (6/1042)	0.776
	Clo 50 WG (2021)	Before IRS	0.0 (0/30)	0.5 (1/215)	1	4.8 (2/42)	3.9 (2/52)	1
		After IRS	1.1 (11/1038)	1.0 (13/1280)	1	5.0 (29/586)	4.4 (68/1544)	0.673

SR: sporozoite rate; %: percentage; A: number of positive head-thoraxes tests; B: total number of heads-thoraxes tested; PM: pirimiphos-methyl; CS: capsule suspensions; WG: Water dispersible granules; Clot + Del: mixture clothianidin 500 g/kg + deltamethrin 62.5 g/kg; Clo 50 WG: clothianidin 50 WG alone

P: p-value of comparisons between control commune and commune under IRS in each department

Overall, there was a reduction in the sporozoite rate after IRS in treated communes compared with control communes. The reduction was statistically significant after IRS inside houses treated in Alibori (P = 0.036) and Donga (P < 0.0001) in 2019 with pirimiphos-methyl 300 CS. Similarly, in the Donga department in 2020, the difference was significant indoors (P = 0.010) with the clothianidin 500 g/kg and deltamethrin 62.5 g/kg mixture (Table 3).

Outside insecticide-treated houses, the sporozoite rate was significantly reduced by pirimiphos-methyl 300 CS in the Donga department. No other differences were observed with other insecticides outside houses sprayed with residual insecticides (Table 3).

### Entomological inoculation rate (EIR) of *An. gambiae* after the 2019, 2020 and 2021 IRS

Data on the entomological inoculation rate (EIR) of *An. gambiae* in the treated area and in the control before and after the IRS (Table 4) reveal a variation in EIR as a function of insecticides in the treated areas before and after the IRS. In the control areas, there was an increase in EIR after IRS despite the change of insecticide. This increase was observed both inside and outside houses (Table 4) in both departments.

**Table 4:** Entomological inoculation rate (EIR) of *An. gambiae s.l*. in the treated area and in the control before and after IRS

Position	Insecticide (Years)	Period	Department of Alibori					Department of Donga				
			Control Commune (Bembèrèkè)		Communes under IRS (Kandi + Gogounou)		P (RR)	Control Commune (Bassila)		Communes under IRS (Djougou + Copargo)		P (RR)
			EIR/night	CI–95%	EIR/night	CI–95%		EIR/night	CI–95%	EIR/night	CI–95%	
Indoors	PM 300 CS (2019)	Before IRS	0.09	[0.08–0.11]	0.00	[0.00–0.00]	< 0.0001 (0.00)	0.16	[0.13–0.18]	0.02	[0.01–0.02]	< 0.0001 (0.13)
		After IRS	0.53	[0.52–0.54]	0.00	[0.00–0.00]	< 0.0001 (0.00)	0.75	[0.73–0.77]	0.03	[0.03–0.03]	< 0.0001 (0.04)
	Clo + Del (2020)	Before IRS	0.00	[0.00–0.01]	0.00	[0.00–0.00]	1 (NaN)	0.00	[0.00–0.02]	0.02	[0.02–0.03]	0.031 (NaN)
		After IRS	0.30	[0.29–0.31]	0.05	[0.05–0.05]	< 0.0001 (0.17)	0.75	[0.73–0.77]	0.20	[0.20–0.21]	< 0.0001 (0.27)
	Clo 50 WG (2021)	Before IRS	0.00	[0.00–0.00]	0.03	[0.03–0.04]	< 0.0001 (NaN)	0.03	[0.02–0.05]	0.01	[0.01–0.02]	< 0.0001 (0.33)
		After IRS	0.54	[0.53–0.55]	0.28	[0.27–0.28]	< 0.0001 (0.52)	0.90	[0.89–0.92]	0.98	[0.97–0.99]	< 0.0001 (1.09)
Outdoors	PM 300 CS (2019)	Before IRS	0.06	[0.05–0.08]	0.02	[0.01–0.02]	< 0.0001 (0.33)	0.03	[0.02–0.05]	0.03	[0.02–0.04]	1 (1.00)
		After IRS	0.22	[0.21–0.22]	0.08	[0.07–0.08]	< 0.0001 (0.36)	0.50	[0.49–0.52]	0.03	[0.03–0.03]	< 0.0001 (0.06)
	Clo + Del (2020)	Before IRS	0.00	[0.00–0.02]	0.00	[0.00–0.00]	1 (NaN)	0.00	[0.00–0.01]	0.02	[0.02–0.03]	0.002 (NaN)
		After IRS	0.25	[0.24–0.26]	0.08	[0.07–0.08]	< 0.0001 (0.32)	0.30	[0.29–0.31]	0.15	[0.15–0.15]	< 0.0001 (0.50)
	Clo 50 WG (2021)	Before IRS	0.00	[0.00–0.01]	0.02	[0.02–0.02]	< 0.0001 (NaN)	0.08	[0.07–0.10]	0.04	[0.03–0.05]	< 0.0001 (0.50)
		After IRS	0.35	[0.35–0.36]	0.21	[0.20–0.21]	< 0.0001 (0.60)	1.09	[1.08–1.11]	1.22	[1.21–1.23]	< 0.0001 (1.12)

EIR: entomological inoculate rate; %: percentage; PM: pirimiphos-methyl; CS: capsule suspensions; WG: Water dispersible granules; Clot + Del: mixture clothianidin 500 g/kg + deltamethrin 62.5 g/kg; Clo 50 WG: clothianidin 50 WG alone; NaN: Not applicable

RR: Rate ratio of EIR per night before and after the implementation of IRS between control commune and communes under IRS within each department, with control commune as the reference

P: p-value of RR methods comparison

In Alibori, a significant decrease in EIR was recorded both inside and outside houses treated after IRS for all three formulations. However, the impact of the insecticide on EIR reduction was most noticeable with pirimiphos-methyl 300 CS, followed by the clothianidin + deltamethrin mixture and finally clothianidin 50 WG alone. Also, the reduction in EIR was higher inside treated houses than outside (Table 4).

In Donga, the findings were similar but of lower magnitude. Furthermore, clothianidin 50 WG induced no reduction in entomological inoculation rates either inside or outside treated houses. Inside treated dwellings, each person could receive 29 infective bites per month, compared with 27 infective bites per month in controls. Outside treated houses, 37 infectious bites per month per person were recorded after IRS, compared with 33 infectious bites per month per person in controls (Table 4).

### Resting density and blood feeding rate of *An. gambiae s.l.*

Overall, the 2019 IRS campaigns with pirimiphos-methyl 300 CS and the 2020 IRS campaigns with clothianidin 500 g/kg + deltamethrin 62.5 g/kg resulted in a significant reduction (p < 0.0001) of *An. gambiae* in treated rooms compared with control rooms (Table 5). However, for the 2021 IRS with clothianidin 50 WG, the resting density of *An*. *gambiae* was similar (p = 0.1503) in treated (1.1) and control (0.9) rooms in Alibori department (Table 6). In the Donga department, the resting density of *An. gambiae* following IRS with clothianidin 50 WG was significantly higher (p = 0.0033) in treated rooms (1.5) than in control rooms (1.0) (Table 5). Pirimiphos-methyl 300 CS performed better than clothianidin 500 g/kg + deltamethrin 62.5 g/kg and clothianidin 50 WG in reducing the resting density of mosquitoes inside treated rooms (Table 6).

**Table 5:** Indoor resting density and blood feeding rates of *An. gambiae s.l.* collected by PSCs method after 2019, 2020 and 2021 IRS intervention in control communes and communes under IRS

Period (Insecticide)	Department	Communes	Nb of room	Nb of *An. gambiae s.l*. collected	Nb of blood feed	Rest density per room			Blood feeding rate	
						Value	RR [95% CI]	P-value	Proportion (%)	P-value
								(Wald)		
After IRS 2019 (PM 300 CS)	Alibori	Bembèrèkè (control)	60	94	75	1.6	1	< 0.0001	79.8	1.00
		Kandi + Gogounou (Under IRS)	120	25	20	0.2	0.13 [0.08–0.21]		80.0	
	Donga	Bassila (control)	30	139	112	4.6	1	< 0.0001	80.6	0.23
		Djougou + Copargo (Under IRS)	120	48	34	0.4	0.09 [0.06–0.12]		70.8	
After IRS 2020 (Clo + Del)	Alibori	Bembèrèkè (control)	60	189	130	3.2	1	< 0.0001	68.8	0.48
		Kandi + Gogounou (Under IRS)	120	102	75	0.9	0.27 [0.21–0.35]		73.5	
	Donga	Bassila (control)	60	201	140	3.4	1	< 0.0001	69.7	0.49
		Djougou + Copargo (Under IRS)	120	118	77	1.0	0.29 [0.23–0.37]		65.3	
After IRS 2021 (Clo 50 WG)	Alibori	Bembèrèkè (control)	80	69	42	0.9	1	0.1503	60.9	0.09
		Kandi + Gogounou (Under IRS)	160	171	125	1.1	1.24 [0.93–1.66]		73.1	
	Donga	Bassila (control)	80	80	71	1.0	1	0.0033	88.8	0.62
		Djougou + Copargo (Under IRS)	160	234	214	1.5	1.46 [1.13–1.91]		91.5	

RR: rate ratio; p (wald): p-value of the Wald test; [95% CI]: 95% confidence interval; %: percentage; PM: pirimiphos-methyl; CS: capsule suspensions; WG: Water dispersible granules; Clot + Del: mixture clothianidin 500 g/kg + deltamethrin 62.5 g/kg; Clo 50WG: clothianidin 50 WG

**Table 6:** Indoor resting density and blood feeding rates of *An. gambiae s.l.* collected by PSCs method after 2019, 2020 and 2021 IRS intervention in communes under IRS

Communes	Period	Nb of room	Nb of *An. gambiae s.l*. collected	Nb of blood feed	Rest density per room			Blood feeding rate	
					Value	RR [95% CI]	P-value	Proportion (%)	P-value
							(Wald)		
Kandi + Gogounou (under IRS in Alibori)	2019 (PM 300CS)	120	25	20	0.2	1	–	80.0	–
	2020 (Clo + Del)	120	102	75	0.9	4.08 [2.61–6.60]	< 0.0001	73.5	0.68
	2021 (Clo 50WG)	160	171	125	1.1	5.13 [3.36–8.15]	< 0.0001	73.1	0.62
Djougou + Copargo (under IRS in Donga)	2019 (PM 300CS)	120	48	34	0.4	1	–	70.8	–
	2020 (Clo + Del)	120	118	77	1.0	2.46 [1.74–3.51]	< 0.0001	65.3	0.61
	2021 (Clo 50WG)	160	234	214	1.5	3.66 [2.67–5.10]	< 0.0001	91.5	0.0002

RR: rate ratio; p (wald): p-value of the Wald test; [95% CI]: 95% confidence interval; %: percentage; PM: pirimiphos-methyl; CS: capsule suspensions; WG: Water dispersible granules; Clot + Del: mixture clothianidin 500 g/kg + deltamethrin 62.5 g/kg; Clo 50WG: clothianidin 50 WG

Overall, blood-feeding rates of *An. gambiae* were high and similar (p > 0.05) in treated (65.3%–91.5%) and control (60.9%–88.8%) rooms (Table 5). No significant difference in blood-feeding rate was observed between the three insecticides in Alibori (Table 6). However, compared with pirimiphos-methyl 300 CS, the blood-feeding rate of mosquitoes inside treated rooms was significantly higher following IRS with clothianidin 50 WG in Donga department (Table 6).

### Parous rate of *An. gambiae* after IRS

Tables 7 and 8 show the impact of IRS on the longevity of *An. gambiae* with pirimiphos-methyl 300 CS, clothianidin 500 g/kg + deltamethrin 62.5 g/kg mixture and clothianidin 50 WG alone. After implementation, the *An. gambiae* parous rate was significantly reduced (p < 0.001) in houses treated with pirimiphos-methyl 300 CS (41.0% to 41.7%) compared with control houses (65.7% to 65.9%) (Table 7). For insecticides containing clothianidin, only the mixture clothianidin 500 g/kg + deltamethrin 62.5 g/kg significantly (p < 0.001) reduced the parous rate in treated houses (44.7% vs. 62.6% in control houses) in Alibori. The reduction in parous rates observed with clothianidin 50 WG between treated and control houses was not statistically significant (Alibori: p = 0.49; Donga: p = 0.17) (Table 7). Comparison of insecticide mortality rates showed that pirimiphos-methyl 300 CS performed very well in reducing the longevity (Table 8) of malaria mosquitoes. It was followed by clothianidin 500 g/kg + deltamethrin 62.5 g/kg mixture and clothianidin 50 WG alone.

**Table 7:** Parous rate of *An. gambiae s.l.* in IRS and control communes after the 2019, 2020 and 2021 IRS campaign

Period (Insecticides)	department	Communes	Nb of *An. gambiae s.l*. dissected	Nb of parous	Parous rate (%)	p-value
2019, after IRS (P M 300CS)	Alibori	Bembèrèkè (control)	551	362	65.7	< 0.0001
		Kandi + Gogounou	427	175	41.0	
	Donga	Bassila (control)	314	207	65.9	< 0.0001
		Djougou + Copargo	470	196	41.7	
2020, after IRS (clo + del)	Alibori	Bembèrèkè (control)	179	112	62.6	0.0004
		Kandi + Gogounou	237	106	44.7	
	Donga	Bassila (control)	247	144	58.3	< 0.0001
		Djougou + Copargo	343	276	80.5	
2021, after IRS (clo 50WG)	Alibori	Bembèrèkè (control)	117	94	80.3	0.49
		Kandi + Gogounou	317	243	76.7	
	Donga	Bassila (control)	77	69	89.6	0.17
		Djougou + Copargo	212	174	82.1	

p-value: comparison of the parous rate of *An. gambiae s.l.* between the treated and control communes (Test used: Chi-square test); Nb: Number; PM: pirimiphos-methyl; CS: capsule suspensions; WG: Water dispersible granules; Clot + Del: mixture clothianidin 500 g/kg + deltamethrin 62.5 g/kg; Clo 50WG: clothianidin 50 WG

**Table 8:** Parous rate of *An. gambiae s.l.* in IRS and control communes after the 2019, 2020 and 2021 IRS campaign

Communes	Period (Insecticides)	Nb of *An. gambiae s.l*. dissected	Nb of parous	Parous rate (%)	p-value
Kandi + Gogounou (under IRS in Alibori)	2019 (PM 300CS)	427	175	41.0	–
	2020 (Clo + Del)	237	106	44.7	0.39
	2021 (Clo 50WG)	317	243	76.7	< 0.0001
Djougou + Copargo (under IRS in Donga)	2019 (PM 300CS)	470	196	41.7	–
	2020 (Clo + Del)	343	276	80.5	< 0.0001
	2021 (Clo 50WG)	212	174	82.1	< 0.0001

p-value: comparison of the parous rate of *An. gambiae* s.l. between the treated (Test used: Chi-square test); Nb: Number; PM: pirimiphos methyl; CS: capsule suspensions; WG: Water dispersible granules; Clot + Del: mixture clothianidin 500 g/kg + deltamethrin 62.5 g/kg; Clo 50WG: clothianidin 50 WG

### Incidence of malaria

**Fig. 3:**
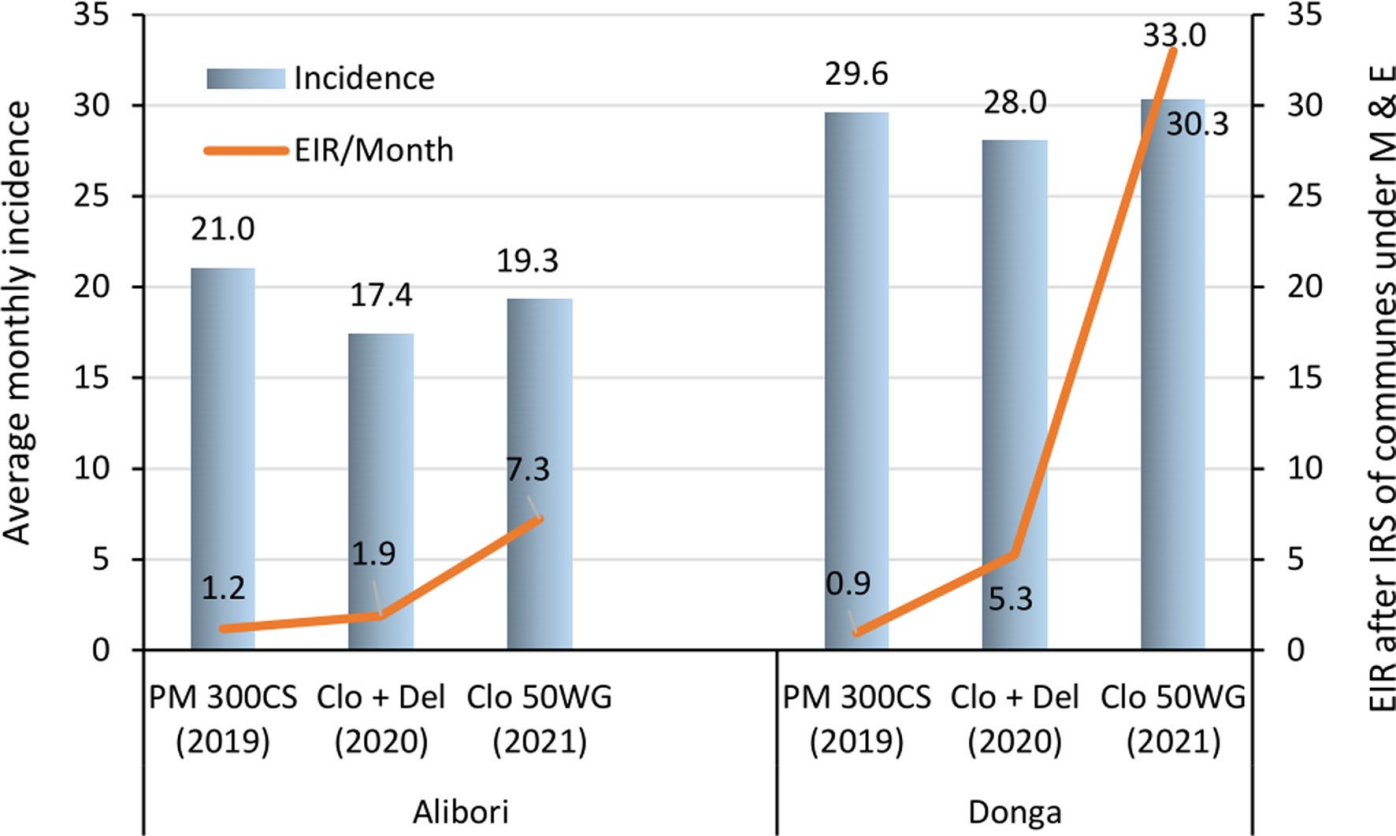
EIR and malaria incidence in communes under IRS monitoring and evaluation

The incidence of malaria in Alibori department was estimated at 17/1000 people per month in 2020 following IRS with the clothianidin 500 g/kg + deltamethrin 62.5 g/kg mixture and at 19/1000 people per month in 2021 following IRS with clothianidin 50 WG versus 21/1000 people per month in 2019 following IRS with pirimiphos-methyl 300 CS. Similar observations were made in Donga with the clothianidin 500 g/kg + deltamethrin 62.5 g/kg mixture, where the incidence of *Plasmodium* was 28/1000 people per month versus 30/1000 people per month following IRS with pirimiphos-methyl 300 CS (Table 9). In the same department, the increase in EIR in 2021 following IRS with the clothianidin 500 g/kg + deltamethrin 62.5 g/kg mixture was associated with an increase in malaria incidence (Fig. 3).

**Table 9:** Average monthly incidence of malaria in communes under IRS monitoring and evaluation, by department and insecticide

IRS communes under M&E	Insecticide used per **year**	Monthly average of new cases	Population	Incidence
Alibori	Pirimiphos-methyl 300 CS (**2019**)	6926	329209	21,03831
	Mixture clothianidin 500 g/kg + deltamethrin 62.5 g/kg (**2020**)	5939	340798	1742675
	Clothianidin 50 WG (**2021**)	6806	352794	1929171
Donga	Pirimiphos-methyl 300 CS (**2019**)	11122	375724	2960152
	Mixture clothianidin 500 g/kg + deltamethrin 62.5 g/kg (**2020**)	10902	388949	28,02938
	Clothianidin 50 WG (**2021**)	12217	402640	3034224

## Discussion

This study was conducted to investigate the difference in malaria transmission indicators (human bite rate, sporozoite rate, entomological inoculation rate, malaria incidence, indoor resting density, blood feeding rate and parous) after IRS with pirimiphos-methyl and clothianidin products. Fluctuations were observed in the abundance of mosquitoes collected according to genus and species per study year. *Culex quinquefasciatus* (59.6%) was the predominant mosquito species collected, followed by *An. gambiae s.l*. (37.6%). Over the 3 years of the study, *Cx. quinquefasciatus* was more abundant in urban, while *An. gambiae s.l.* was generally predominant in rural. The abundance of *Cx. quinquefasciatus* may be linked to its ability to lay eggs and develop in a variety of natural and artificial larval habitats [24]. Of the 8 species of *Anopheles* collected, *An*. *gambiae s.l*., *An*. *funestus* and *An*. *nili* were identified as malaria vectors. The *An*. *gambiae* complex was omnipresent throughout the study area, accounting for 98.6% (18,683/18,940) of all *Anopheles* collected. The *An*. *funestus* group was the second most abundant [1% (190/18,940)] followed by *An*. *nili* [0.03% (5/18,940)]. This predominance of *An*. *gambiae s.l.* was previously reported in northern Benin [25–27] in Kandi, Atacora and Donga and Alibori, respectively. Molecular characterization of *An*. *gambiae s.l*. showed that *An*. *gambiae s.s.* (52.9%), *An*. *coluzzii* (46.4%) and *An*. *arabiensis* (0.8%) are the members of this complex in the two departments studied. These results are similar to those of Salako et al*.* [27].

The reduction in the rate of bites inside treated houses compared with control houses suggests the complementary prevention represented by IRS in endemic areas where LLINs are used as a preventive intervention. Previously, Akogbéto et al. [28], have shown high rates of *An*. *gambiae* bites on humans inside houses using only LLINs as a means of prevention.

The results of study indicate a greater reduction in human-vector contact, sporozoite rate (SR) and entomological inoculation rate (EIR) of *An. gambiae* both inside and outside homes sprayed with pirimiphos-methyl 300 CS in the Alibori and Donga departments. There was also a moderate reduction in all three indicators inside homes sprayed with the clothianidin 500 g/kg + deltamethrin 62.5 g/kg mixture. In the case of clothianidin 50 WG alone, both indoor and outdoor biting rates and sporozoite infections remained high after IRS. These results confirm the efficacy of pirimiphos-methyl 300 CS used in indoor residual spraying (IRS) for the elimination and control of pyrethroid-resistant malaria vectors. In northern Benin and Kenya, the findings of work by Akogbéto et al*.* [22], Salako et al*.* [21] and Abong'o et al*.* [23] have demonstrated the positive impact of pirimiphos-methyl 300 CS IRS in reducing entomological indicators of malaria transmission. The 10-month residual efficacy of pirimiphos-methyl 300 CS in Kenya in 2020 [29], led not only to a drastic reduction in the density of *An*. *funestus*, the main malaria vector, but also to a reduction in the number of malaria cases recorded at health facilities. The positive effect of IRS with pirimiphos-methyl 300 CS on the longevity of malaria vectors at rest and outside treated dwellings in Alibori and Donga departments results from the rapid action [30] of the insecticide. The reduction in malaria transmission with clothianidin 50 WG alone and the clothianidin 500 g/kg + deltamethrin 62.5 g/kg PID mixture against pyrethroid-resistant mosquitoes was higher indoors but the effects were not statistically significant. Clothianidin-based insecticides used in IRS have a slow lethal effect on mosquitoes.

When the *Anopheles* field strain comes into contact with clothianidin, it can survive for up to 120 h [15, 31, 32]. The results of studies by Hoppé et al*.* [34] and Lees et al*.* [35] diverge on the efficacy of clothianidin. Hoppé et al*.* [34] reported low insecticidal activity of clothianidin against *Aedes aegypti*, a mosquito species responsible for the transmission of diseases, such as dengue and Zika virus infection. Lees et al*.* [35] found clothianidin to be sufficiently effective against mosquitoes, particularly *Anopheles*, in tarsal contact tests. These contradictory results may be due to several factors, such as research methodologies, mosquito species studied, clothianidin application conditions, and possibly other unknown factors.

The resting density of *An*. *gambiae* inside sprayed houses is significantly lower than in unsprayed houses in 2019 with the use of pirimiphos-methyl 300 CS in IRS and in 2020 with the mixture clothianidin 500 g/kg + deltamethrin 62.5 g/kg throughout the study area. However, in 2021, the opposite situation was observed, with more *An*. *gambiae* resting in houses sprayed with clothianidin 50 WG alone compared to control houses. This result is similar to that of Agossa et al*.* [37] at Covè, where the exophily induced by clothianidin 50 WG alone was relatively low as clothianidin does not have excito-repulsive and irritant properties.

The high feeding rates observed in treated dwellings may be explained by the anthropophilic behaviour of *An*. *gambiae* which seeks its blood meal first before resting. These results are similar to those obtained in other studies [25, 28, 38–41]. During the first 2 h after sunset, *An*. *gambiae* females feed on blood [33, 42] when household residents are engaged in domestic activities or watching television or radio. Also, *An*. *gambiae* may also take a blood meal at dusk [33] when household members leave their LLINs for prayer and other domestic activities such as cooking. After feeding, the females of *An*. *gambiae* go to rest on the various supports hung on the walls sprayed with insecticide to avoid contact with the insecticide.

The low rate of parity observed in homes sprayed with pirimiphos-methyl confirms the high insecticidal effect of this compound and a reduction in adult longevity. These results confirm those of Salako et al. [27]. However, no significant difference was observed with clothianidin 50 WG alone. This may be due to its slow action [33] as mosquitoes may be able to complete a full gonotrophic cycle before succumbing to the insecticide. However, the lack of difference in sporozoite rates suggests that this insecticide had minimal impact on mosquito longevity in Benin.

Despite the increasing EIR in 2020 and 2021 with the use of clothianidin 500 g/kg + deltamethrin 62.5 g/kg mixture and clothianidin 50 WG alone in IRS, malaria incidence was slightly reduced in Alibori in both years, though only in 2020 in Donga. This reduction in incidence through the decline in the number of new malaria cases recorded is probably explained by IRS and the national distribution and use of LLINs which were distributed through a mass campaign in 2020 intended to cover all households counted throughout the country [36]. This slight reduction in new malaria cases could also be attributed to the confinement measures introduced in response to COVID-19. This measure engendered fear and a change in behaviour among the population with regard to access to health services. For fear of being diagnosed positive for COVID-19 and being separated from their families, some patients would prefer to stay at home to receive treatment. For others, health centres no longer seemed to be appropriate options during this period, as they were perceived as places at risk of contamination by COVID-19.

However, it should be noted that clothianidin's poor performance in reducing entomological indicators of malaria transmission could be linked to other factors such as climate and intervention coverage [15].

## Conclusion

Entomological monitoring and evaluation of clothianidin alone and the clothianidin + deltamethrin mixture for indoor residual spraying in Alibori and Donga showed no significant overall impact on indicators such as SR, HBR, EIR, indoor resting density and blood-feeding rate of *An. gambiae* in contrast to pirimiphos-methyl.

Overall, indoor residual spraying with clothianidin-based insecticides did not further reduce vector populations and malaria transmission indicators, despite the longer residual efficacy compared with the shorter residual efficacy of pirimiphos-methyl-based products.

## Data Availability

The data used and/or analysed in this study are available from the corresponding author on reasonable request.

## Abbreviations

IRS: Indoor residual spraying
WHO: World health organization
ETI: Entomological transmission indicators
HBR: Human biting rate
SR: Sporozoite rate
EIR: Entomological inoculation rate
HLC: Human landing catch
PSC: Pyrethrum spray catch
CREC: Centre de recherche entomologique de cotonou
NMCP: National malaria control program
CS: Capsulated suspension
WP: Wettable powders
WG: Water-dispersible granules

## References

[1] WHO. World malaria report 2017. Geneva: World Health Organization; 2017 (https://www.who.int/publications/i/item/9789241565523).

[2] Cibulskis RE, Alonso P, Aponte J, Aregawi M, Barrette A, Bergeron L (2016). Global progress 2000–2015 and future challenges. Infect Dis Poverty.

[3] WHO. World Health Organization (2015). Indoor residual spraying: an operational manual for indoor residual spraying (IRS) for malaria transmission control and elimination –.

[4] Akogbéto MC, Dagnon F, Aïkpon R, Ossé R, Salako AS, Ahogni I (2020). Lessons learned, challenges and outlooks for decision-making after a decade of experience monitoring the impact of indoor residual spraying in Benin. West Africa Malar J.

[5] Sharp BL, Ridl FC, Govender D, Kuklinski J, Kleinschmidt I (2007). Malaria vector control by indoor residual insecticide spraying on the tropical island of Bioko. Equatorial Guinea Malar J.

[6] Fullman N, Burstein R, Lim SS, Medlin C, Gakidou E (2013). Nets, spray or both? The effectiveness of insecticide-treated nets and indoor residual spraying in reducing malaria morbidity and child mortality in sub-Saharan Africa. Malar J.

[7] Kamya MR, Kakuru A, Muhindo M, Arinaitwe E, Nankabirwa JI, Rek J (2020). The impact of control interventions on malaria burden in young children in a historically high-transmission district of Uganda: a pooled analysis of cohort studies from 2007 to 2018. Am J Trop Med Hyg.

[8] Coleman S, Dadzie SK, Seyoum A, Yihdego Y, Mumba P, Dengela D (2017). A reduction in malaria transmission intensity in Northern Ghana after 7 years of indoor residual spraying. Malar J.

[9] Protopopoff N, Wright A, West PA, Tigererwa R, Mosha PW, Kisinza, et al. Combination of insecticide treated nets and indoor residual spraying in northern Tanzania provides additional reduction in vector population density and malaria transmission rates compared to insecticide treated nets alone: a randomised control trial. PLoS ONE. 2015;10:e0142671. 10.1371/journal.pone.0142671 PMC4646432 26569492

[10] WHO. The evaluation process for vector control products. Geneva, World Health Organization, 2018.

[11] Hemingway J, Ranson H, Magill A, Kolaczinski J, Fornadel C, Gimnig J (2016). Averting a malaria disaster: will insecticide resistance derail malaria control?. Lancet.

[12] OMS, Stratégie technique mondiale de lutte contre le paludisme 2016–2030. Genève, Organisation mondiale de la Santé. 2015.

[13] WASH. Groupe Régional WASH, Afrique de l’Ouest et Centrale. Version du 7 Avril 2017 https://www.humanitarianresponse.info/en/operations/west-and-central-africa/water-sanitation-hygiene

[14] World Health Organization. List of WHO Prequalified Vector Control Products. Geneva, World Health Organization, 2023. https://extranet.who.int/pqweb/vector-control-products/prequalified-product-list.

[15] Odjo EM, Salako AS, Padonou GG, Yovogan B, Adoha CJ, Adjottin B (2023). What can be learned from the residual efficacy of three formulations of insecticides (pirimiphos-methyl, clothianidin and deltamethrin mixture, and clothianidin alone) in large-scale in community trial in North Benin, West Africa?. Malar J.

[16] Aïkpon R, Afoukou C, Hounpkatin B, Eclou DD, Cyaka Y, Egwu E (2020). Digitalized mass distribution campaign of insecticide-treated nets (ITNs) in the particular context of Covid-19 pandemic in Benin: challenges and lessons learned. Malar J.

[17] NASA. Power data Access Viewer: https://power.larc.nasa.gov/data-access-viewer/

[18] Gillies MT, De Meillon B (1968). The Anophelinae of Africa south of the Sahara. S Afr Inst Med Res.

[19] Santolamazza F, Mancini E, Simard F, Qi Y, Tu Z, della Torre A.  (2008). Insertion polymorphisms of SINE200 retrotransposons within speciation islands of *Anopheles gambiae* molecular forms. Malar J.

[20] Detinova TS, Gillies MT (1964). Observations on the determination of the age composition and epidemiological importance of populations of *Anopheles gambiae* Giles and *Anopheles funestus* Giles in Tanganyika. Bull World Health Organ.

[21] Wirtz RA, Zavala F, Charoenvit Y, Campbell GH, Burkot TR, Schneider I (1987). Comparative testing of monoclonal antibodies against *Plasmodium falciparum* sporozoites for ELISA development. Bull World Health Organ.

[22] OMS. Entomologie du paludisme et lutte antivectorielle, Guide du participant. Genève, Organisation mondiale de la Santé. 2014.

[23] Mulla MS, Norland RL, Fanara DM, Darwezeh HA, McKean DW (1971). Control of chironomid midges in recreational lakes. J Econ Entomol.

[24] Gil MF, Fassolari M, Battaglia ME, Beron CM (2021). *Culex quinquefasciatus* larvae development arrested when fed on *Neochloris aquatica*. PLoS Negl Trop Dis.

[25] Gnanguenon V, Govoetchan R, Agossa FR, Osse R, Oke-Agbo F, Azondekon R (2014). Transmission patterns of *Plasmodium falciparum* by *Anopheles gambiae* in Benin. Malar J.

[26] Aïkpon R, Ossè R, Govoetchan R, Sovi A, Oké-Agbo F, Akogbéto MC (2013). Entomological baseline data on malaria transmission and susceptibility of *Anopheles gambiae* to insecticides in preparation for indoor residual spraying (IRS) in Atacora, (Benin). J Parasitol Vector Biol.

[27] Salako AS, Dagnon F, Sovi A, Padonou GG, Aïkpon R, Ahogni I (2019). Efficacy of Actellic 300 CS-based indoor residual spraying on key entomological indicators of malaria transmission in Alibori and Donga, two regions of northern Benin. Parasit Vectors.

[28] Akogbéto MC, Salako AS, Dagnon F, Aïkpon R, Kouletio M, Sovi A (2018). Blood feeding behaviour comparison and contribution of *Anopheles coluzzii* and *Anopheles gambiae*, two sibling species living in sympatry, to malaria transmission in Alibori and Donga region, northern Benin. West Africa Malar J.

[29] Abong’o B, Gimnig JE, Torr SJ, Longman B, Omoke D, Muchoki M, et al. Impact of indoor residual spraying with pirimiphos-methyl (Actellic 300CS) on entomological indicators of transmission and malaria case burden in Migori Country, western Kenya. Sci Rep. 2020;10:4518 10.1038/s41598-020-61350-2 PMC7066154 32161302

[30] Mhadhbi L, Beiras R (2012). Acute toxicity of seven selected pesticides (Alachlor, Atrazine, Dieldrin, Diuron, Pirimiphos-Methyl, Chlorpyrifos, Diazinon) to the marine fish (Turbot, *Psetta maxima*). Water Air Soil Pollut.

[31] Kweka E, Mahande A, Ouma J, Karanja W, Msangi S, Temba V (2018). Novel indoor residual spray insecticide with extended mortality effect: a case of SumiShield 50WG against wild resistant populations of *Anopheles arabiensis* in Northern Tanzania. Glob Health Sci Pract.

[32] Oxborough RM, Seyoum A, Yihdego Y, Dabire R, Gnanguenon V, Wat’senga F, et al. Susceptibility testing of *Anopheles malaria* vectors with the neonicotinoid insecticide clothianidin; results from 16 African countries, in preparation for indoor residual spraying with new insecticide formulations. Malar J. 2019;18:264. 10.1186/s12936-019-2888-6 PMC6670198 31370898

[33] Carnevale P, Bosseno MF, Molinier M, Lancien J, Le Pont F, Zoulani A (1979). Étude du cycle gonotrophique d’*Anopheles gambiae* (Diptera, Culicidae) (Giles, 1902) en zone de forêt dégradée d’Afrique Centrale. Cahiers ORSTOM, sér Ent Méd Parasitol.

[34] Hoppé M, Hueter OF, Bywater A, Wege P, Maienfisch P (2016). Evaluation of commercial agrochemicals as new tools for malaria vector control. Chimia (Aarau).

[35] Lees R, Praulins G, Davies R, Brown F, Parsons G, White A (2019). A testing cascade to identify repurposed insecticides for next-generation vector control tools: screening a panel of chemistries with novel modes of action against a malaria vector. Gates Open Res.

[36] WHO. World malaria report. Geneva: World Health Organization. 2021

[37] Agossa FR, Padonou GG, Koukpo CZ, Zola-Sahossi J, Azondekon R, Akuoko OK, et al. Efficacy of a novel mode of action of an indoor residual spraying product, SumiShield^(R)^ 50WG against susceptible and resistant populations of *Anopheles gambiae* (s.l.) in Benin, West Africa. Parasit Vectors. 2018;11:293. 10.1186/s13071-018-2869-6 PMC5946391 29747684

[38] Agossa FR, Aïkpon R, Azondékon R, Govoetchan R, Padonnou GG, Oussou O (2014). Efficacy of various insecticides recommended for indoor residual spraying: pirimiphos methyl, potential alternative to bendiocarb for pyrethroid resistance management in Benin, West Africa. Trans R Soc Trop Med Hyg.

[39] Ototo EN, Mbigi JP, Wanjala CL, Zhou G, Githeko AK, Yan G (2015). Surveillance of malaria vector population density and biting behavior in Western Kenya. Malar J.

[40] Padonou GG, Gbedjissi G, Yadouleton A, Azondekon R, Ossé R, Oussou O, et al. Decreased proportions of indoor feeding and endophily in *Anopheles gambiae* s.l. populations following the indoor residual spraying and insecticide-treated net interventions in Benin (West Africa). Parasit Vectors. 2012;5:262. 10.1186/1756-3305-5-262 PMC3523976 23151270

[41] Aïkpon R, Sèzonlin M, Tokponon F, Okè M, Oussou O, Oké-Agbo F (2014). Good performances but short lasting efficacy of Actellic 50 EC Indoor Residual Spraying (IRS) on malaria transmission in Benin. West Africa Parasit Vectors.

[42] Brun LO (1973). Contribution à l’étude biologique et écologique des vecteurs majeurs de paludisme en Afrique de l’Ouest.

